# Effect of Caffeine on Attention and Alertness Measured in a Home-Setting, Using Web-Based Cognition Tests

**DOI:** 10.2196/resprot.6727

**Published:** 2017-09-07

**Authors:** Wilrike J Pasman, Ruud Boessen, Yoni Donner, Nard Clabbers, André Boorsma

**Affiliations:** ^1^ TNO Department of Microbiology and Systems Biology Zeist Netherlands; ^2^ TNO Department of Risk Analysis for Products In Development Zeist Netherlands; ^3^ Stanford University Department of Computer Sciences Stanford, CA United States

**Keywords:** caffeine, at-home testing, cognition, EFSA claim

## Abstract

**Background:**

There is an increasing interest among nutritional researchers to perform lifestyle and nutritional intervention studies in a home setting instead of testing subjects in a clinical unit. The term used in other disciplines is ‘ecological validity’ stressing a realistic situation. This becomes more and more feasible because devices and self-tests that enable such studies are more commonly available. Here, we present such a study in which we reproduced the effect of caffeine on attention and alertness in an at-home setting.

**Objective:**

The study was aimed to reproduce the effect of caffeine on attention and alertness using a Web-based study environment of subjects, at home, performing different Web-based cognition tests.

**Methods:**

The study was designed as a randomized, placebo-controlled, double-blind, crossover study. Subjects were provided with coffee sachets (2 with and 2 without caffeine). They were also provided with a written instruction of the test days. Healthy volunteers consumed a cup of coffee after an overnight fast. Each intervention was repeated once. Before and 1 hour after coffee consumption subjects performed Web-based cognitive performance tests at home, which measured alertness and attention, established by 3 computerized tests provided by QuantifiedMind. Each test was performed for 5 minutes.

**Results:**

Web-based recruitment was fast and efficient. Within 2 weeks, 102 subjects applied, of whom 70 were eligible. Of the 66 subjects who started the study, 53 completed all 4 test sessions (80%), indicating that they were able to perform the do it yourself tests, at home, correctly. The Go-No Go cognition test performed at home showed the same significant improvement in reaction time with caffeine as found in controlled studies in a metabolic ward (*P*=.02). For coding and N-back the second block was performed approximately 10% faster. No effect was seen on correctness.

**Conclusions:**

The study showed that the effects of caffeine consumption on a cognition test in an at-home setting revealed similar results as in a controlled setting. The Go-No Go test applied showed improved results after caffeine intake, similar as seen in clinical trials. This type of study is a fast, reliable, economical, and easy way to demonstrate effectiveness of a supplement and is rapidly becoming a viable alternative for the classical randomized control trial to evaluate life style and nutritional interventions.

**Trial Registration:**

Clinicaltrials.gov NCT02061982; https://clinicaltrials.gov/ct2/show/NCT02061982 (Archived by WebCite at https://clinicaltrials.gov/ct2/show/NCT02061982)

## Introduction

There is increasing interest in the scientific research in measuring health parameters in a real-life setting instead of using a clinical unit, facilitated by using eHealth and mHealth. In medicine, mHealth for example is used in type 2 diabetics resulting in improved monitoring and diabetes management of patients themselves [1].

Many tests for measuring health parameters are commonly available in drug stores as well as Web-based, enabling self-measurement. In addition, many consumer devices are available that, with increasing reliability, measure health parameters. Calibration of these methods, uploading the data of these devices, privacy, and security of data health portals, are currently all important aspects to make these methods applicable for consumer health science.

Because more people have mobile phones, in which all sorts of applications are available, it is used for research too. Apps replace face-to-face contact [2] and enable testing in free-living subjects. At-home data collection could provide a better picture of real-life situations. Web-based data collection was reviewed by Swan [3] as an important emerging complement to clinical trials. Addition of a more standardized, organized design is recommended for more reliable data than by various crowdsourced data collections.

A recent systematic review [4] investigated the diversity and effectiveness of all sorts of digital interventions. Variability in type of interventions, definitions, outcome measures, and reporting of results of the different studies made interpretation of the results difficult. This finding stresses that a more guided manner of conducting these studies is beneficial. When structured in a clear protocol, this could be introduced for performing a randomized intervention trial in a real-life setting by consumers themselves. If successful, the costs for a clinical trial are no longer a substantial part of the budget for research. Moreover, the parameters of interest will be measured within the real-life setting where the tested product (or intervention) is ultimately aimed at being used by consumers at home. Examination of the effects in the real-life environment is known as ‘ecological validation’ [5].

Studies that are used for supporting nutrition and health claims are especially interesting for this approach. The European Food Safety Authority (EFSA) is verifying the scientific substantiation of the submitted claims on food products. Most of the studies that are used for supporting such claims have been conducted in a clinical setting; however, for these claims a real-life situation would be more suitable. In other words, the ecological validity of such studies can be questioned. Testing the effect of food products using materials and in the setting that is the best approximate of the real world, will also produce much more robust claims. An effect present in a less controlled environment as in real life, measured while there is more variation present, does really exist (less false positives). Although ecological validity of neuropsychological tests is under debate with respect to everyday cognitive skills as dependent upon the population tested, the approach used in the study, the experimenter conducting the tests, as well as the environment [5]; the controlled ‘office-based tests’ (lab condition) on cognitive function alone do not give a complete picture of behavior. Real-world observations, or tests performed in a real-world setting improve executive function assessment [6].

In the present study, we focus on the EFSA claim of caffeine, in which it is stated that 75 to 150 mg of caffeine increased alertness and attention [7]. In the EFSA document, numerous controlled studies are described in which an effect of caffeine on cognition tests was found. The control in these studies are mostly related to the sex and age of subjects, habitual smoking and drinking habits (regular coffee consumption), order of activities during the test days, the number of cognitive tests, fasting state, activities allowed in the test session, food and drinking rules prior to the tests, duration of caffeine deprivation, and so on [8-12].

Brice and Smith [8], who studied single and multiple coffee consumptions, found reduced reaction times. Already as a control of habitual coffee consumption, 1 cup of coffee hourly, the study was designed to compare one large single dose of caffeine versus multiple small doses of caffeine consumption. The test days were controlled for start time, number of doses provided, timing of consumption, and types of subjects (male, young, non-smoking). It was found that both regimes showed increased alertness and improved performance of cognition tasks. The authors therefore concluded that findings from large, single-dose studies could be applicable for normal consumption effect [8].

The level of deprivation of caffeine and smoking was studied by Fine [9], who concluded that due to caffeine deprivation, high-caffeine consumers showed poorer results on cognition tests. Restriction of caffeine and nicotine prior to testing is a normal standardization procedure, which may in itself affect the outcome [9]. But others found that even in subjects minimally deprived of caffeine with a low dose of 75 mg of caffeine, a performance enhancing effect was found in the lab 1 hour after consumption [10]. So cognitive performance improvement was found, even when no caffeine withdrawal is present [10].

In a study were habitual coffee consumers versus nonconsumers were tested, similar improvements were seen for caffeine consumption; both showed faster reaction times and improved mood [11].

Improvement of reaction time was also found in a study using a dose range of 32 to 256 mg of caffeine, in which for all caffeine consumption tests improved performance (more correct answers) and a 5% faster reaction time was present [12]. Control was present for the provision of test dose, caffeine consumption throughout the experiment, the order of the tasks, and the baseline practice sessions. It is concluded that although laboratory tasks, the objective benefits of caffeine could be useful in other settings, like automobile driving (although also simulated in a lab).

Establishing effects on attention and alertness require cognitive tests on reaction time and vigilance. These tests are available in various formats and sources, but are commonly only available on a local computer or network. Quantification of alertness and cognition in an at-home setting, relevant tests require Internet-based and scientifically reliable applications. Quantified Mind is a project in the United States [13] that provides a wide range of cognition tests. Data collection on response time may however be a source of variation in itself due to differences in laptops and browsers used [14].

To study the feasibility of a real-world setting, a relatively simple study intervention should be chosen, of which the effect is well known and acknowledged, like the effect of caffeine on alertness and attention. Moreover, study subjects can perform a caffeine intervention at home with relatively minor effort on their daily habits. The required dose to be able to demonstrate an effect is within the normal range of daily use (at least 75-mg caffeine is required). This dosage corresponds with a cup of regular coffee and was used in the present study to test the at-home setting.

We therefore designed a randomized, double-blind study to repeat a classical randomized control trial as conducted for caffeine on cognitive function (EFSA claim), in which parameters are measured in a real-life, at-home setting instead of in a metabolic ward with a controlled setting.

The objective of the present study was to examine the reproducibility of caffeine on attention and alertness of subjects using a Web-based study environment with different cognition tests at home without contact with the subjects compared with results from controlled, clinical studies.

## Methods

### Subject Recruitment

In October 2013, subjects were recruited via 2 main Internet sites: Facebook and FoodLog, a popular food blog for (professional) people interested in food (research). Additional people were recruited via LinkedIn or by word of mouth. In total, 102 subjects showed their interest. The interested subjects were provided a study information document by email. There were 74 subjects who wanted to participate and were sent an informed consent form and a health and lifestyle questionnaire (hard copy). Subjects were eligible when healthy (according to questionnaire), ≥18 years of age, able to perform tests on the computer/laptop, moderate caffeine users, had no mental disorder or used medication for this, participated voluntary, and sent a signed informed consent form. After completion and return of these documents, we had 70 eligible subjects (see Multimedia Appendix 1).

The EFSA caffeine claim document [7] showed that the caffeine tests were conducted with at least 35 subjects. To compensate for possible dropout of approximately 20%, we wanted to include at least 50 subjects.

### Study Design

The study was designed as a randomized, placebo-controlled, double-blind, crossover study.

The subjects participated in 5 study days: 1 training day and 4 test days on which the interventions with placebo (decaf) or caffeine were measured, consuming 1 treatment per test day.

A complete training day preceded the tests to have the main learning effects in this first session, and start with all subjects at a rather similar learning level independent of their acquired testing skills. All 3 cognition tests were conducted similarly as on a normal test day (duration, type, and level of difficulty of the tests). The order of the cognition test was always the same (Coding test; Go-No Go; N-back).

### Conduct of the Study

The eligible subjects were provided with 4 sachets of coffee (2 with decaf [coded A] and 2 with caffeine [coded B]) so they could prepare the coffee at home. The order of using the sachets was mentioned in a letter. Together with the coffee sachets, an instruction document was sent on how to prepare the coffee and the order of actions of the tests days with respect to timing of drinking and testing.

Subjects performed the tests in the morning after an overnight fast. After filling in a wellbeing questionnaire and the sleepiness scale, the subjects performed 3 cognition tests on his/her own personal computer or laptop. Then preparation and drinking of coffee was scheduled, and after 1 hour, the computer tests were conducted again. During the total period of the test (~1.5 hour), the subjects were not allowed to eat, drink, or smoke anything except for the coffee.

Information was provided to allow each test subject to perform the Web-based cognition tests. Website, user name, and passwords were provided and details of the tests were explained. Subjects with login problems could call or mail our helpdesk service.

Analysis of the provided coffee revealed that the caffeinated sachets contained 85 mg of caffeine. In the decaffeinated sachets 3 mg of caffeine was present.

The study was performed according to guidelines in the Declaration of Helsinki, and the institutional review board of Brabant, the Netherlands approved all procedures (NL 45382.028.13). Registration was done prior to the start of the study [NCT02061982]. The study was performed in November to December 2013.

### Data Collection

Effects on alertness and attention were measured using 3 computerized cognitive tests provided by Quantified Mind. The 3 tests were performed by the subjects at t=0 (baseline, prior to coffee consumption) and t=1 hour in one go. Each test was performed for 5 minutes. During the training session the participants were instructed to get acquainted with the tasks. The 3 cognition tests used are described in Multimedia Appendix 2.

### Statistics

Each subject performed 2 placebo and 2 caffeine-coupled tests. Counterbalancing was applied to account for potential carry-over effects between subsequent test days as a result of learning, boredom, fatigue, and so on. This was done by dividing the full 4-day experiment into 2, 2-day blocks in which every subject received both decaf and caffeine. This resulted in 2 randomization schemes (ABAB and BABA), which were both assigned to one-half of the study population each. As a result, potential carry-over effects were equally distributed over both treatment conditions.

Each cognition test revealed 2 data sets, reaction time and correctness of the responses. Separate analyses were performed on the reaction times and on the dichotomous true/false outcomes. Reaction times were analyzed using a repeated-measures analysis of the variance (ANOVA) with treatment (ie, caffeine or decaf), block (first or second 2 days of testing), and the treatment × block interaction, as well as a random intercept for subject to account for correlations between repeated measures collected from individual subjects. True/false outcomes were analyzed with a repeated measures logistic regression model that also included treatment, block, and the treatment × block interaction, and a random intercept for subject.

The residual plots of the data were checked for normal distribution of the data. For the reaction time data, the curves were not normally distributed and had to be log transformed first. The model assumptions were met enabling the use of ANOVA. The models provided estimates of the overall means per treatment condition and block as well as the interaction between these two. Differences between means were deemed significant when the corresponding 2-sided *P* value was below .05. All statistical analyses were conducted using SAS 9.2.

## Results

### Baseline Characteristics

Of the 70 subjects who started the study, we obtained a complete data set of all test sessions of 53 subjects. There were 17 subjects (25%) who started the study but were unable to perform all tests days completely. Of the 53 subjects, 77% (41/53) were female and 23% (12/53) were male, with a mean age of 36 years (standard deviation, 14 years). In Table 1, a description of the baseline characteristics of the subjects is presented. The subjects had a high education level and showed a healthy lifestyle (low smoking rate; moderate alcohol consumption; physically active). Subjects were used to consuming coffee and tea regularly.

Of the cognition sessions performed by the participants, 10% to 20% were not completed. Some missing data was therefore present for A and B, but the model was able to use these incomplete data.

Responses with a reaction time under 200 ms and above 4000 ms were excluded from the analysis; the first were considered anticipatory, the second caused by something other than effortful cognitive processing. In total, these excluded responses accounted for 1.3% of all data points.

### Cognition Tests

**Figure 1:**
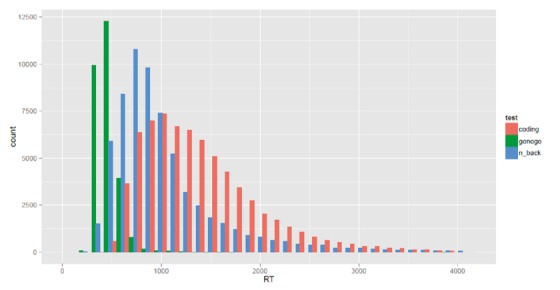
Reaction time frequencies (in ms) of the 3 cognition tests of the subjects: Coding (red), Go-NoGo (green), N-back (blue) test.

**Figure 2:**
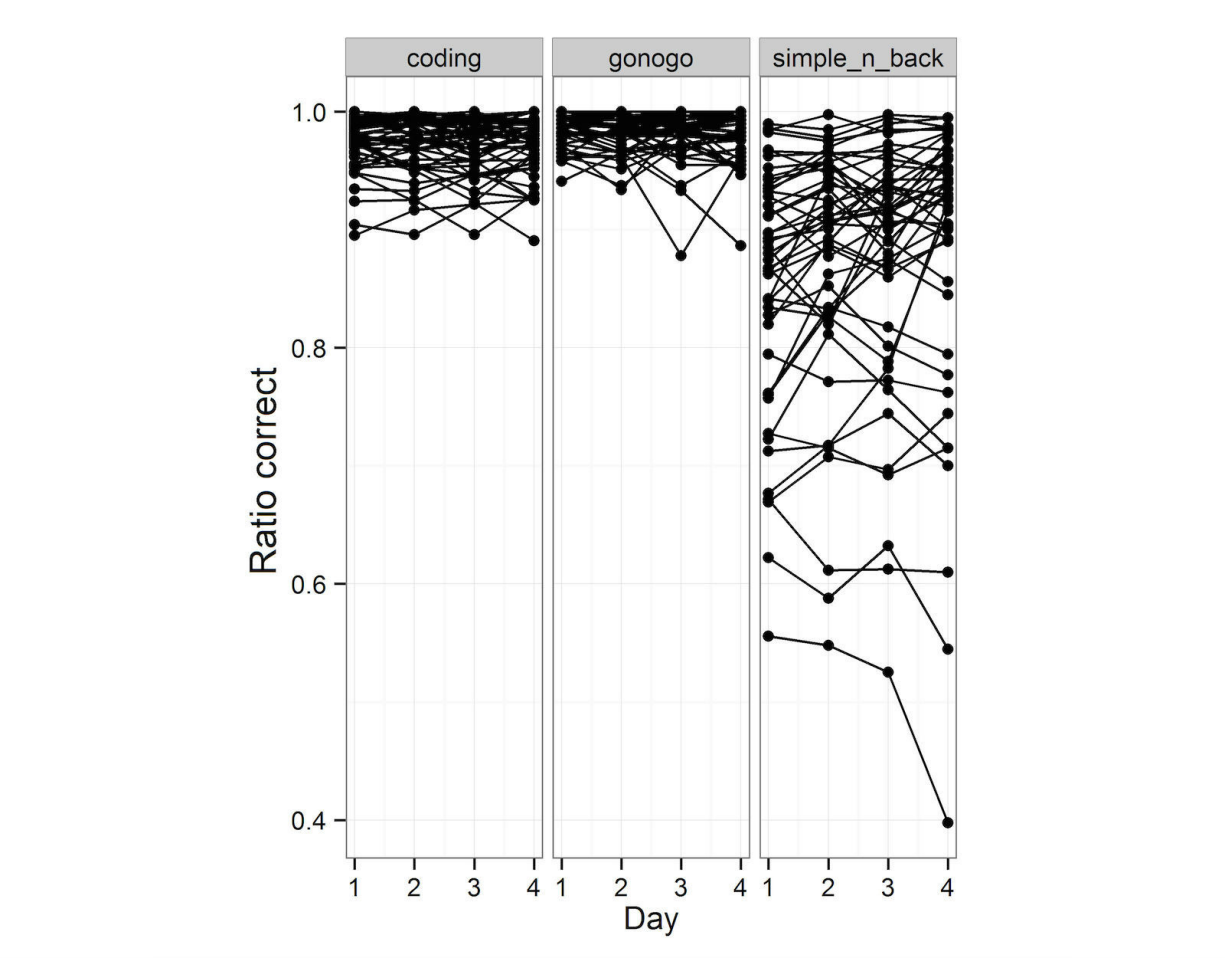
Ratio correct responses of total responses of the three cognitive tests on the four test days: Coding, Go-NoGo, N-back tests.

The 3 cognition tests used, revealed different reaction time frequency spectra, stressing the different types of cognition performance required for the test. The Go-No Go showed the fastest reaction time; Coding and N-back testing required more time for answering (see Figure 1 for reaction time spectrum of the tests). The ratio of correct and incorrect responses also illustrates the various levels of difficulty of the 3 tests (see Figure 2).

**Table 1:** Baseline characteristics of subjects (n=53).

Parameters and characteristics			n (%)
**Age (years)**			
	All subjects 36 ± 14		53 (100)
	Men: 36 ± 14		12 (23)
	Women: 36 ± 14		41 (77)
**Recruitment**			
	Facebook		16 (30)
	FoodLog.nl		21 (40)
	Other media		16 (30)
**Daily physical activity: Dutch activity norm (.5 hours activity/day)**			
	Below Dutch norm		14 (26)
	Met the Dutch norm		39 (74)
**Alcohol consumption**			
	No		10 (19)
	**Yes**		43 (81)
		1–7 consumptions/week	36 (68)
		8–14 consumptions/week	5 (9)
		15–21 consumptions/week	2 (4)
**Smoking habit**			
	Yes		6 (11)
	**No**		47 (89)
		Just stopped	4 (8)
		Quit	15 (28)
		Never	28 (53)
**Habitual coffee consumption**			
	No		0 (0)
	**Yes**		53 (100)
		<7 cups/week	16 (30)
		7-14 cups/week	18 (34)
		>14 cups/week	19 (36)
**Habitual tea consumption**			
	No		12 (23)
	**Yes**		41 (77)
		<7 cups/week	17 (32)
		7-14 cups/week	12 (23)
		>14 cups/week	12 (23)

#### Reaction Time

The reaction time results of the cognition tests performed 1 hour before consumption of the provided coffee sachets (baseline) and after coffee consumption (intervention) is presented in Table 2.

A clear treatment effect of caffeine was present for the Go-No Go test after coffee consumption (*P*<.001), resulting in a faster response after caffeine intake. For the Coding and N-back tests this was not seen. For Coding, a block effect was present (*P*<.001) and for N-back an interaction effect was found (*P*=.006).

The baseline cognition data collected before coffee consumption did not show a treatment effect. An interaction was found for Coding (*P*=.001) and for N-back (*P*=.007). For Go-No Go, a block effect was seen (*P*=.005).

#### Correct Responses

In Table 3, the correct response index of the cognition tests performed 1 hour before consumption of the provided coffee sachets (baseline) and after coffee consumption (intervention) are presented. The data collected at baseline revealed a treatment effect (*P*=.044) for Coding, and a block effect for the N-back test (*P*<.001). The intervention of coffee consumption showed small reduction in correct answers for the Go-No Go test (*P*=.004) for the second block. Improvement of correct answers in the second block was seen for the N-back test (*P*<.001).

**Table 2:** Reaction time (in ms) at baseline and after coffee consumption for decaf and caffeine conditions.^a^

Test and treatment		Baseline			Intervention		
		Block 1	Block 2	*P* value	Block 1	Block 2	*P* value
**Coding**							
	Decaf	1506 ± 613	1413 ± 571	<.001^b^	1483 ± 592	1405 ± 566	<.001^e^
	Caffeine	1506 ± 621	1401 ± 587		1465 ± 572	1397 ± 577	<.001^e^
**Go-No Go**							
	Decaf	436 ± 137	442 ± 117	.005^c^	426 ± 102	434 ± 109	<.001^f^
	Caffeine	446 ± 121	423 ± 102		420 ± 95	418 ±1 03	
**N-Back**							
	Decaf	1093 ± 618	925 ± 506	.007^d^	1013 ± 570	877 ± 487	.006^g^
	Caffeine	1089 ± 606	985 ± 567		1005 ± 580	917 ± 525	

^a^At baseline the coffee is not yet consumed, but it reflects the condition for that test day. Both coffee conditions were repeated and shown as block 1 and block 2 data.

^b^Treatment × block interaction Coding Intervention.

^c^Block effect Go-No Go Intervention.

^d^Treatment × block interaction for N-back; especially between block 1 and block 2 for both treatments.

^e^Block effect for Intervention.

^f^Treatment effect of intervention.

^g^Interaction effect of intervention.

**Table 3:** Correct response index at baseline and after coffee consumption for decaf and caffeine conditions.^a^

Test and treatment		Baseline			Intervention		
		Block 1	Block 2	Statistics	Block 1	Block 2	Statistics
**Coding**							
	Decaf	0.967 ± 0.177	0.966 ± 0.181	.044^b^	0.973 ± 0.162	0.967 ± 0.178	
	Caffeine	0.967 ± 0.179	0.972 ± 0.164	.044^b^	0.967 ± 0.178	0.967 ± 0.177	
**Go-No Go**							
	Decaf	0.981 ± 0.136	0.981 ± 0.136		0.985 ± 0.121	0.979 ± 0.142	.004^d^
	Caffeine	0.981 ± 0.136	0.981 ± 0.137		0.986 ± 0.118	0.981 ± 0.133	.004^d^
**N-Back**							
	Decaf	0.859 ± 0.348	0.877 ± 0.328	<.001^c^	0.875 ± 0.330	0.887 ± 0.316	<.001^e^
	Caffeine	0.862 ± 0.345	0.876 ± 0.329	<.001^c^	0.876 ± 0.329	0.879 ± 0.326	<.001^e^

^a^At baseline the coffee is not yet consumed, but it reflects the condition for that test day. Both coffee conditions were repeated and shown as block 1 and block 2 data.

^b^Treatment effect Coding baseline.

^c^Block effect for N-back baseline.

^d^Block effect for intervention.

^e^Block effect for intervention.

## Discussion

### Principal Results

For the first time to our knowledge, in the present study we describe the conduct of a human intervention study design (via use of the Internet) to test the effect of caffeine on cognition (EFSA claim) in a real-world setting. Instead of using a clinical setting for conduction of the studies [8-12], participants were tested in their home environment. This study may be of importance for food companies to enable testing of their products in a more natural environment. The effect of caffeine on cognition was measured using 3 different tests, requesting different cognitive functions (attention, alertness, [visual] memory). We found a significant treatment effect in the ‘at-home condition’, what may be interesting for ecological validity of the tested (food) product.

Our main finding was a faster reaction time in the Go-No Go test, as a result of caffeine consumption prior to the test. The test resulted in a significant finding even in an environment (at home) where more variation in the test conditions and performance measurements was anticipated. The significant difference in reaction time of 10 ms of decaf versus caffeine condition may not be meaningful in real life, but was similar to that found in the controlled condition [8]. The reduced reaction time of 330 to 320 ms [8] was already faster than in the present study, but a similar reduction was present. A very strict time protocol was used in the study of Brice and Smith [8], but was not precisely known in the present study because subjects perform the tests themselves at home. Smith and Rogers saw a similar improvement in reaction time as well (±510 ms for placebo and ±490 ms for 100-mg caffeine) [15]. The more pronounced effect found in the latter study might be a result of the fact that both habitual caffeine consumers, as well as caffeine abstainers were included in the study. In our study, only subjects habituated to coffee and tea consumption were included. This may explain the more pronounced effect in reaction time, because the effect in abstainers may be increased, although there is still debate whether abstainers show increased effects [15].

For this type of study, it is important to stress the amount of data available. The tests contain multiple tasks and are mostly performed for at least 5 minutes, resulting in thousands of data points for all subjects together. This explains the significant finding, although still a large standard deviation was present. This was in line with well-controlled lab trials [12,15,16].

The main difference from the well-controlled clinical trials [8-12] was the level of control of the conduct of the test day. Timing of the tests performed, product preparation, compliance, and conduct of the tests are performed according to protocol supervised by a research nurse able to correct the subject. At home, more variation will be present. In the present study, subjects were provided with an instruction how to prepare the drink, the timing of consumption, and performance of the tests; otherwise, too much variation may result in less interpretable results in these type of studies [4].

Internet and laptop differences may be a source of variation as well. In the study, we used the website of QuantifiedMind for standardized testing. So the software used was controlled. However, there may have been variation in the browser used, the central processor unit of the laptop [14]. This may give rise to additional variation in reaction time as was recently discussed. Restricted inclusion of type of laptop and browser should be beneficial for reduction in variation, but may be difficult with respect to recruitment of subjects.

The variation at home was exactly the item reported in the evaluation questionnaire by the participants; some deviations from the protocol were mentioned (minor time differences, nonfasting state, order of activities deviated). This means that there was indeed more variation present at home than in a controlled metabolic ward trial environment. This indicates that for this type of study less power is present and more subjects need to be included to find significant effects. The number of subjects in controlled caffeine studies was 18 [8] and 24 [10]; our study was conducted with twice as many subjects, just to increase the power with this variation in outcomes and have ecological validity.

For the different cognition tests, we found that the significantly reduced reaction time for the second block illustrates that subjects were still on a learning curve at the test days. More training sessions than the 1 test day session and the training practices at each test day before the actual tests were performed, may provide better test conditions to measure differences due to a treatment intervention. The finding that a learning effect is still present and affecting reaction time, stresses the importance of training sessions in these types of studies. The design for an at-home cognition study should therefore contain multiple test days so that training will not affect the outcome of the test anymore or contain tests with less training effects (simple, straightforward tests).

Due to the randomized and balanced order of treatment in the study the learning effect is not affecting the outcome of the study.

### Web-Based Recruitment and Subject Population

The websites used for recruiting subjects, resulted in a fast recruitment (Facebook and FoodLog) as was reported before [17]. Highly motivated subjects with an interest in food research showed interest in participation. Both websites revealed clearly highly educated subjects. The high education level and the healthy lifestyle of this group of subjects (see Table 1: low alcohol intake, low smoking percentage, high activity level; good computer skills) stressed the selectivity of our population. Nicholl [4] also recently reported that participants were predominantly female, white, well educated, and middle aged, and thus the wider applicability of digital self-management interventions remains uncertain. This is of importance for the introduction of the usability of these at-home tests. It may be not be applicable for the general population.

Also, our subject population showed a predominance of women in the study of 77% (41/53) versus 23% (12/53) men. The mean age of 36 ± 14 years further showed that we did not have a young group of subjects, both factors were in line with Nicholl [4] and indicate that we cannot generalize our results to the whole population.

### At-Home Testing

To study the feasibility of the real-world setting, a relatively simple study intervention should be chosen, of which effect is well known. In addition, the study measurements should be able to be performed by volunteers at home when subjects conduct the protocol alone and unguided. At-home testing further requires computer-skilled subjects, a portal with adequate Web-based tests/questionnaires, and a helpdesk at the research site. The study procedures performed by the subjects need to be straightforward; products to be consumed must be sent in time and clearly coded.

In the study evaluation the subjects stressed that the cognition tests were easy to implement in their daily activity schedule. The at-home study design therefore had a high compliance score. However, not all cognition tests used are applicable: the coding test was difficult and needed too much explanation, and is therefore not suitable to implement in an ‘at-home setting’.

### Strengths and Limitations

Many strengths can be identified in our study. The study was designed as a randomized controlled trial. The subjects were provided with clear instructions on how to perform the tests themselves at home. The decaf and caffeine coffee sachets looked similar and were coded and were both tested twice. Intake of which coffee sachet was consumed, was checked via the Internet. Some compliance questions were asked before the start of the tests. Cognitive baseline testing was included to examine day-to-day variation. These are all aspects of well-controlled studies.

There were some limitations in the study as well. In the evaluation of the study, it became clear that some subjects performed the study differently than prescribed (nonfasting state; time deviations; order of activities deviated). Therefore, it is known that these tests contained more variation than completely controlled clinical studies. Improvement of the prescribed conduct of the study may be visualization; besides written information, provision of instruction films may help a correct conduct of the tests or study at home.

The set up at home is therefore less suitable for strict or complicated study protocols. Easy research questions with simple read outs for data collection and adequate, tailored instructions are essential. A reduction in variation of conducting the study may be achieved by a Web-based check prior to the start of the tests to examine understanding of the protocol and conduct of the tests.

Another inherent limitation in this type of study is reliability of the selection criteria due to absence of face-to-face contact or even blood or physiological data because subjects are recruited via the Internet. The screening form can be manipulated and this again will result in more noise/variation of the subject data.

The fact that no control of the conduct is present, other than the electronic data obtained, is a clear limitation. Some control was present (electronic data, code of coffee sachet consumed), but subjects could manipulate the tests. Collection of a saliva sample in order to encourage compliance with the test instruction, but not analyzing the sample as done by others [15] may limit this factor. This strategy may stimulate subjects to perform the study as correct as possible.

Implementation of webcam use to improve or monitor compliance may affect privacy too much, resulting in fewer subjects willing to participate. This may also be a technologic issue for subjects unable to upload movies.

A study design based on Internet websites requires skills with respect to computers and the Internet. Based on starting numbers in our study, 25% (17/70) of the subjects were not able to complete the tests or found the study too time consuming. The website built was not easy for subjects, because of the double login; after the login to the Do It Yourself (DIY) caffeine study, they needed to login on QuantifiedMind website separately. Of course, this can be improved both by better websites and better evaluation of computer skills prior to study performance.

Further study should also be done on the variation present in these types of studies. Now we have one large cloud of variation, but well-controlled examination of all kind of factors influencing the variation may provide insight into the amount of effect: the differences in time of testing, preparation, environment, people (children) present, fasting, eating the evening before, activities done before, and so on. Control of these factors may show what elements are of real importance affecting the outcome for at home testing.

### Conclusions

In the present study, it is shown that a DIY study conducted at home is a valuable alternative for well-controlled studies.

On the Go-No Go cognition test, the DIY caffeine study presented showed a similar faster response as found in controlled studies in a metabolic ward as used in the EFSA claim for caffeine. Not all cognitive tests are sensitive enough or suitable for at-home testing. The learning effect present with cognitive tests, stress the importance of training sessions prior to actual testing. The limited control and the variation in conduct by subjects themselves at home, stress the use of the DIY design for simple, straightforward research questions, and a clear instruction protocol. The easier recruitment and the lower costs for conducting, make this type of design and attractive addition to the current randomized control trial portfolio.

## Appendix

Multimedia Appendix 1 Subject inclusion.

Multimedia Appendix 2 Cognition tests.

Multimedia Appendix 3 CONSORT-EHEALTH checklist (v1.6.1).

## Abbreviations

ANOVA: analysis of the variance
DIY: do it yourself
EFSA: European Food Safety Authority
