# Supplementary figures and images for: Effect of Caffeine on Attention and Alertness Measured in a Home-Setting, Using Web-Based Cognition Tests
Source: JMIR Res Protoc. 2017 Sep 7;6(9):e169. doi: 10.2196/resprot.6727 (PMC5608989; doi:10.2196/resprot.6727)

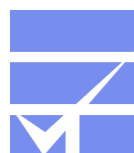

# CONSORT

TRANSPARENT REPORTING of TRIALS

## Assessed for eligibilityCONSORT 2010 Flow Diagram

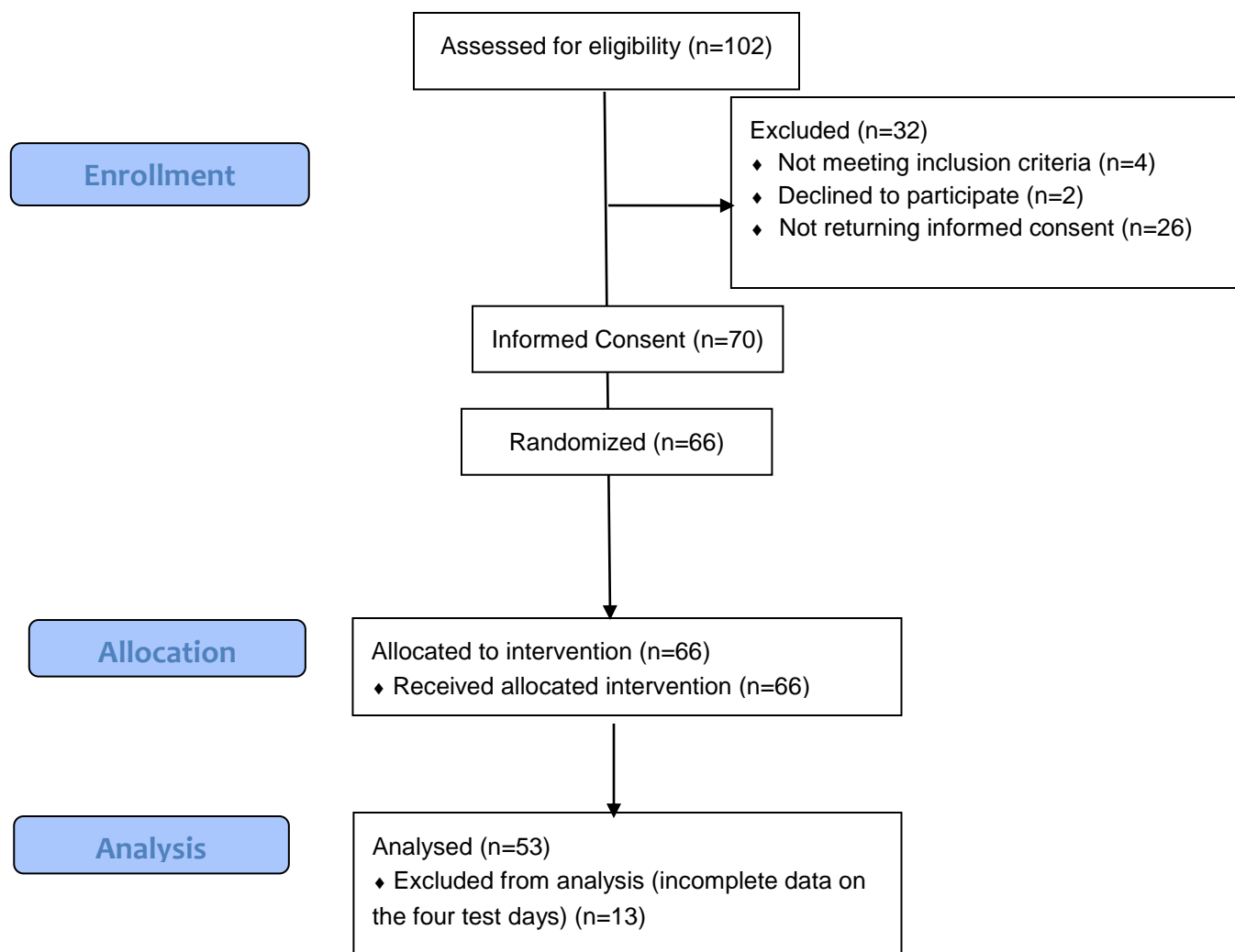

Supplement: Multimedia Appendix 1 [file resprot_v6i9e169_app1.pdf]
