# Supplementary material for: Effect of Caffeine on Attention and Alertness Measured in a Home-Setting, Using Web-Based Cognition Tests
Source: JMIR Res Protoc. 2017 Sep 7;6(9):e169. doi: 10.2196/resprot.6727 (PMC5608989; doi:10.2196/resprot.6727)

Supplementary file Cognition tests

Go / No-Go test

The test “Go/ No-Go” assesses sustained attention, inhibition, and impulsivity by measuring whether and how quickly a user presses a button in response to a target stimulus. Target stimuli are small white squares that appear at the top of a black screen. There are also non-target stimuli, small white squares that appear at the bottom of the black screen, which the user is instructed to ignore. Stimuli are presented one at a time, and statistics are kept on how often a target stimulus is ignored by the user, how often the user presses the button in response to a non-target stimulus, how quickly the user presses the button in response to target stimuli, and how often the user presses the button too soon for target stimuli. This combination of measurements assessed sustained attention--the ability of a user to pay attention in spite of boredom and inactivity, and inhibition control-the ability to react quickly and accurately to target stimuli while managing to avoid reacting to non-targets or reacting too early to targets.

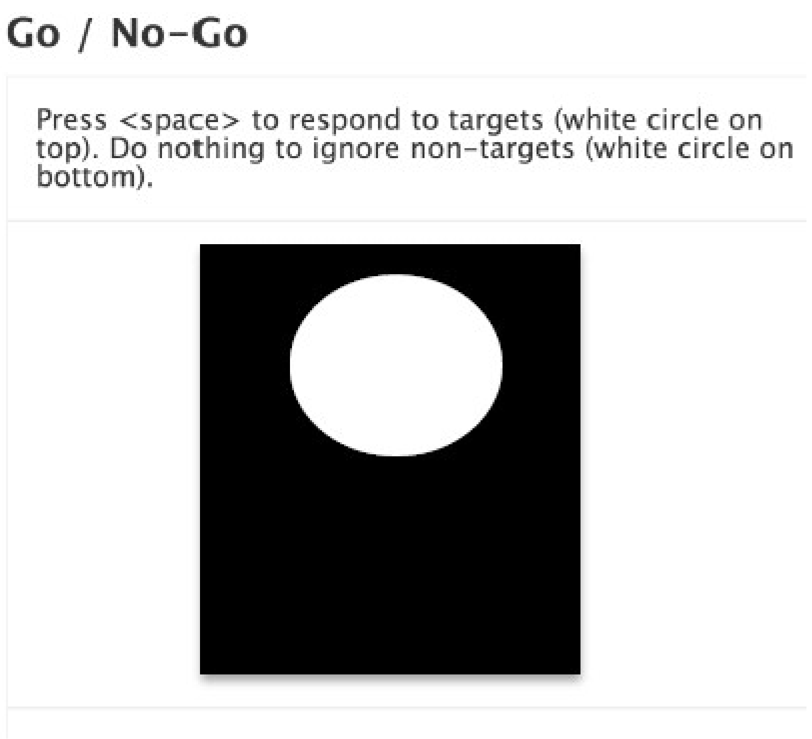

N-back test

Working memory and higher cognitive functions such as fluid intelligence were tested with the use of N-back. In an N-back test, a series of stimuli is presented, and the participant is asked to recall which stimulus was presented a specified number (n) back in the sequence. Stimuli can be numbers, letters, or pictures. If n is one, the correct response is the stimulus presented immediately prior to the last one. If n is two (like example below), the correct response is the stimulus presented two prior to the last one.

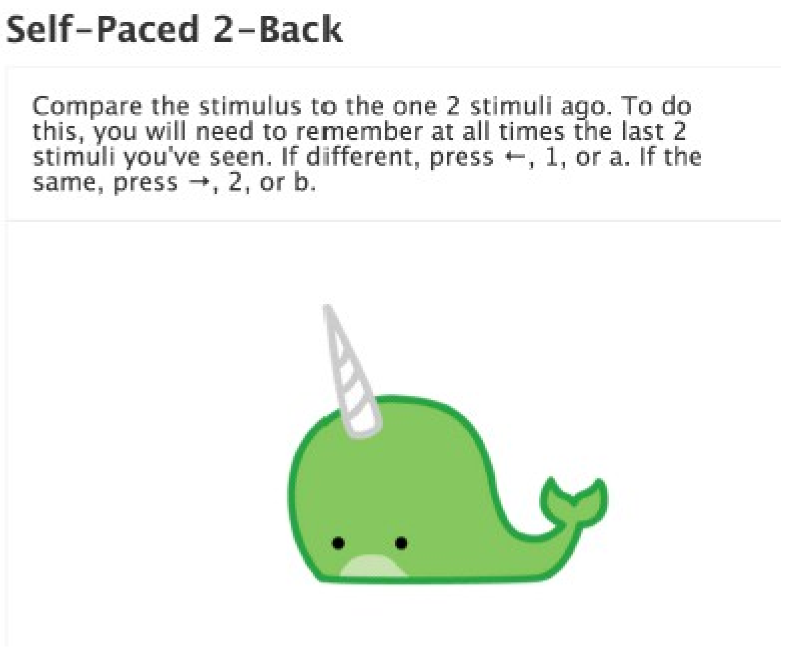

Coding test

A mixed visual memory, attention, and acuity test is called “Coding”. Takers of this test must match the presently displayed image to one of the one images displayed above under each of nine numbers. Good visual memory improves performance on this task by making the answering process more efficient, but visual memory is not strictly necessary, since the user always has the option to directly compare the displayed image with the answer choices.

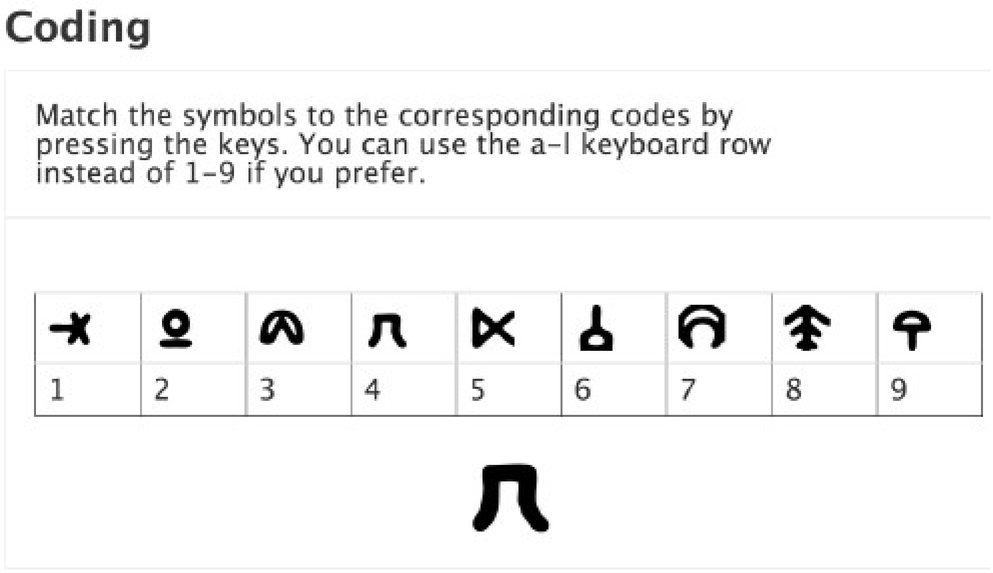

Supplement: Multimedia Appendix 2 [file resprot_v6i9e169_app2.pdf]
